# Supplementary figures and images for: Light-sheet microscopy for everyone? Experience of building an OpenSPIM to study flatworm development
Source: BMC Dev Biol. 2016 Jun 30;16:22. doi: 10.1186/s12861-016-0122-0 (PMC4929743; doi:10.1186/s12861-016-0122-0)

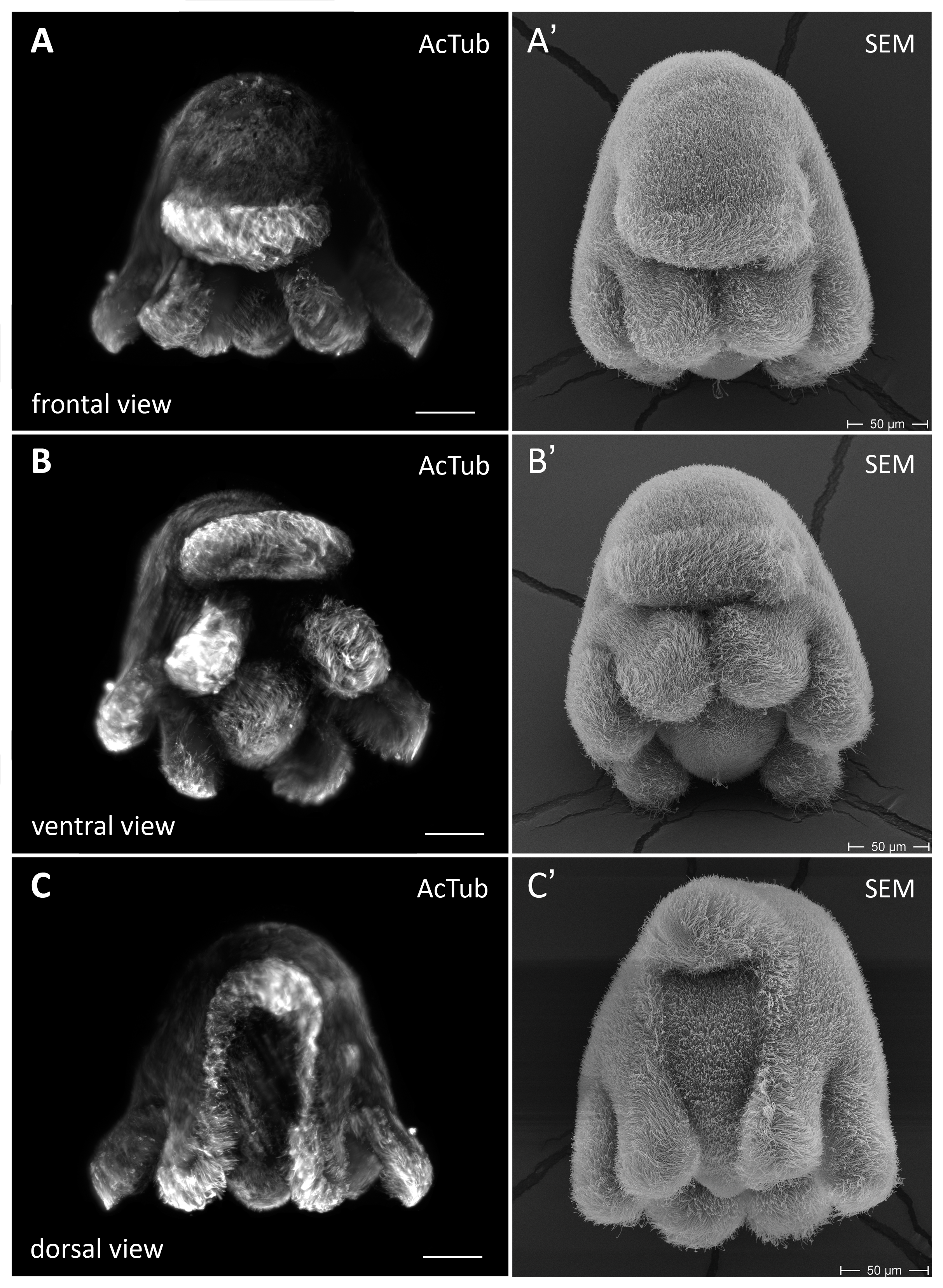

Supplement: Additional file 1: Figure S1. — Images (maximum projections) of fixed Müller’s larvae stained with Acetylated tubulin and captured with our OpenSPIM images show a clear resemblance to scanning electron microscopy images of similar stage larvae. (TIF 7028 kb) [file 12861_2016_122_MOESM1_ESM.tif]

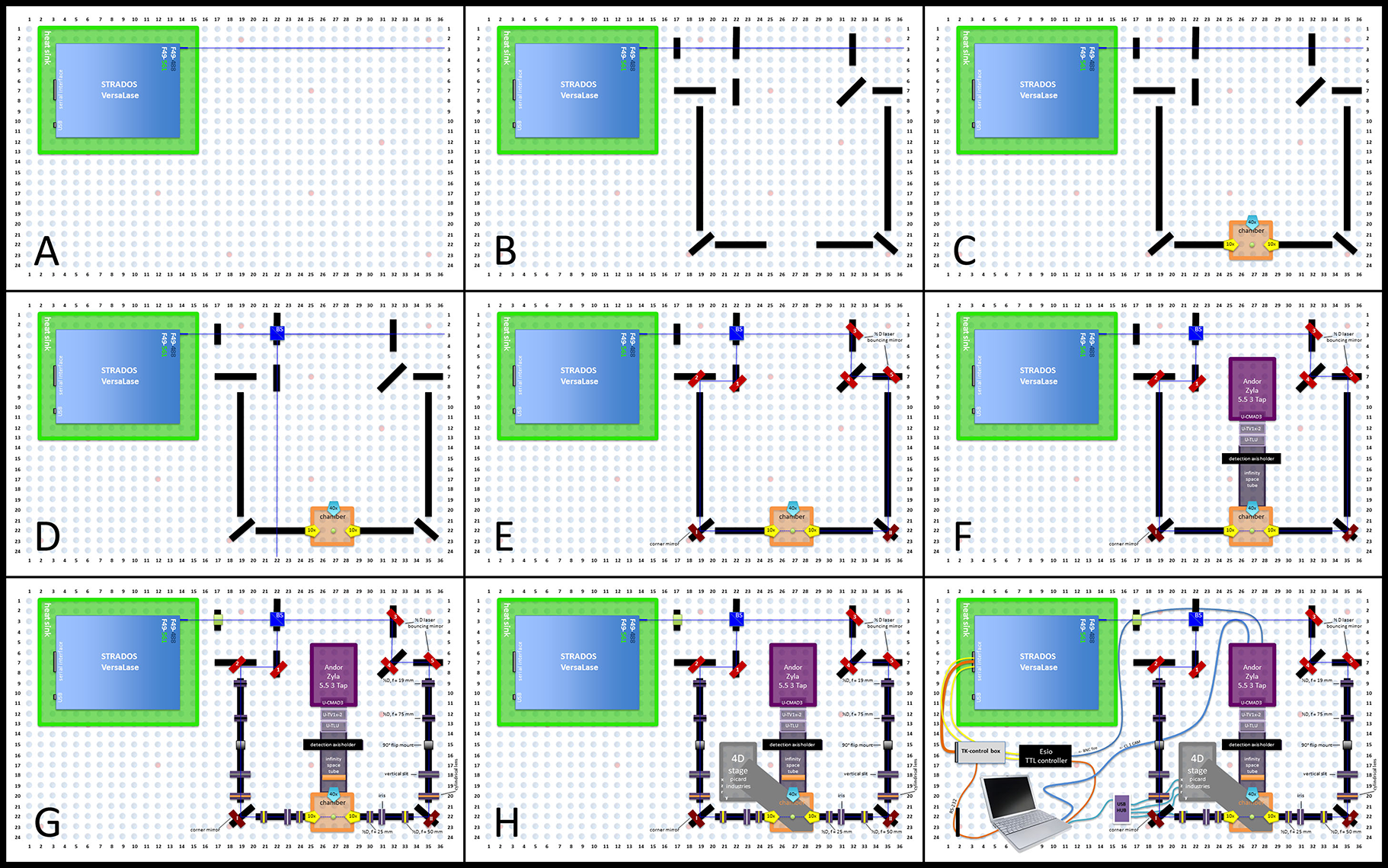

Supplement: Additional file 5: Figure S2. — Schematic assembly of the OpenSPIM; (A) Step 1 - Installation of breadboard feet; Step 2 - Installation of laser heatsink and fixation of laser system (VersaLase) on top (B) Step 3 - Cutting and installation of rail system onto the optical breadboard (C) Step 4 - Installation of pre-assembled acquisition chamber (D) Step 5 - Installation of the beam splitter (E) Step 6 - Installation of all corner and laser reflecting mirrors (F) Step 7 - Installation of detection axis holder, infinity space tube, camera and its corresponding connection adapter units to the infinity space tube (U-CMAD3, U-TV1x-2 and U-TLU) (G) Step 8 - Installation of optical elements (beam expanders, telescope); Step 9 - Installation of clean-up and emission filters (H) Step 10 - Installation of Picard 4D stage on its correct position (I) Step 11 - Plugging in the controller boxes (Esio TTL controller box & VersaLase control box), VersaLase, Camera, USB 4D-stage and connecting them up with the acquisition computer. (TIF 2639 kb) [file 12861_2016_122_MOESM5_ESM.tif]

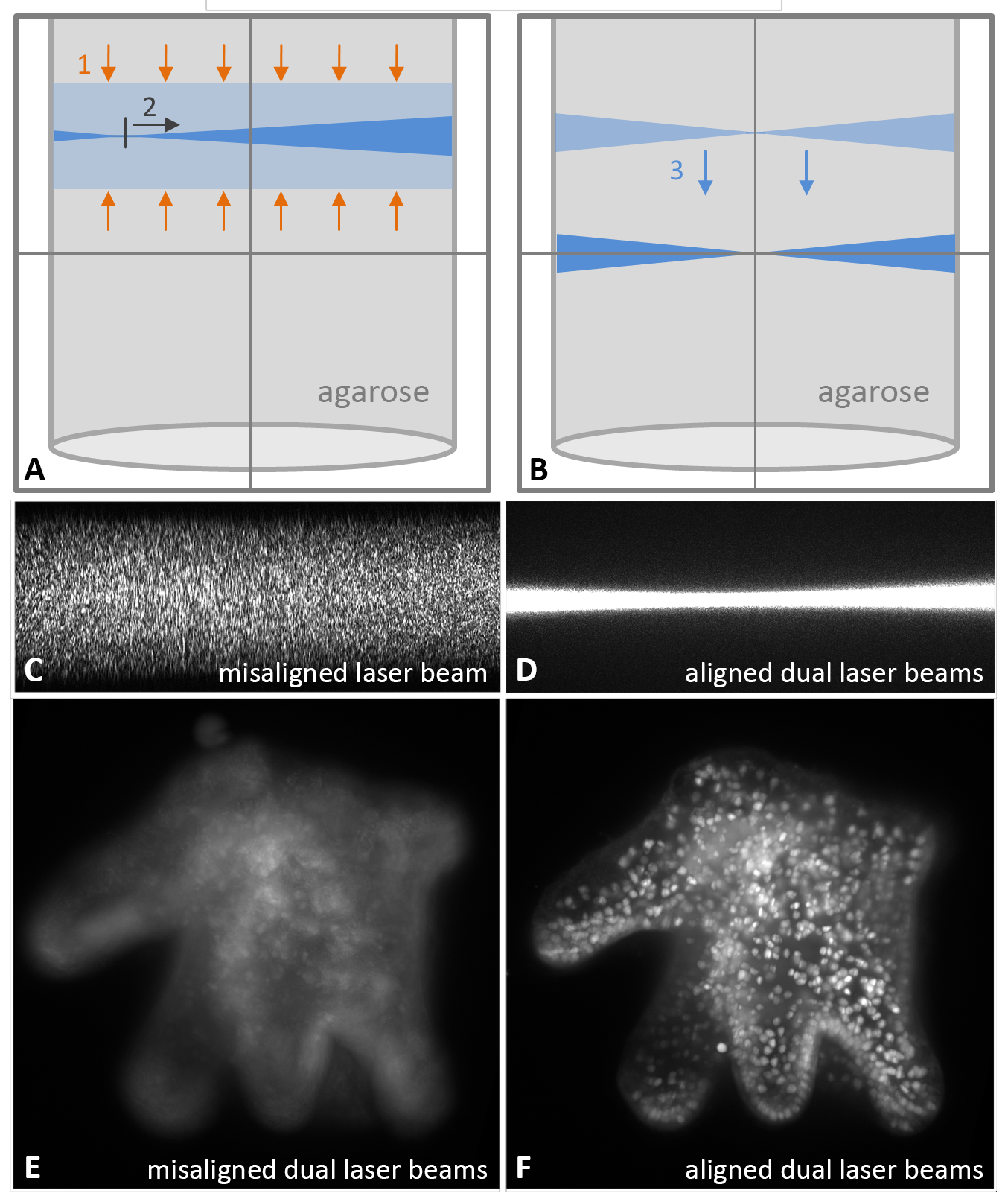

Supplement: Additional file 6: Figure S3. — A and B Schematic drawing of laser beam visualized on agarose hanging from above into the water filled acquisition chamber, Also seen in A and B are three alignment steps of the laser beam (1-3); C and D Actual misaligned and aligned laser beams visualized on agarose by removing emission filters and cylindrical lenses; E and F SPIM images (maximum projections) acquired with misaligned and aligned laser beams. The initially visible fuzzy beam is indicated by a bright blue horizontal stripe in between orange arrows. This coarse beam is then brought into focus with the detection objective (step1) and therefore appears as a much thinner laser beam indicated by a blue horizontal stripe in an hourglass-like shape. Note that in this example the focal point of the beam is at this point still shifted to the left (vertical grey line) and need further adjustments (step2). B The laser beam is shifted from the top to a central position within the field of view (step3). (TIF 8563 kb) [file 12861_2016_122_MOESM6_ESM.tif]
