# Supplementary material for: Light-sheet microscopy for everyone? Experience of building an OpenSPIM to study flatworm development
Source: BMC Dev Biol. 2016 Jun 30;16:22. doi: 10.1186/s12861-016-0122-0 (PMC4929743; doi:10.1186/s12861-016-0122-0)
Supplement: Additional file 4: — Supplementary Methods. (DOCX 17 kb) [file 12861_2016_122_MOESM4_ESM.docx]

**Light-sheet microscopy for everyone?**

**Experience of building an OpenSPIM to study flatworm development**

Supplementary Material

Johannes Girstmair^1^, Anne Zakrzewski^1^, François Lapraz^1,2^, Mette Handberg-Thorsager^3^, Pavel Tomancak^3^, Peter Gabriel Pitrone^3^, Fraser Simpson^1^, Maximilian J. Telford^1^ *

^1^ Department of Genetics, Evolution and Environment, University College London, London, WC1E 6BT United Kingdom

^2^ CNRS, CBD UMR5547, Université de Toulouse, UPS, Centre de Biologie du Développement, Bâtiment 4R3, 118 Route de Narbonne, 31062 Toulouse, France

^3^ Max Planck Institute of Molecular Cell Biology and Genetics, Pfotenhauerstr. 108, 01307 Dresden, Germany

* Author for correspondence: m.telford@ucl.ac.uk

**Supplementary Methods**

**OpenSPIM assembly in 14 simplified steps**

Assembly steps (see also Additional file 5: Figure S2): **Step 1:** Installation of breadboard feet onto the optical breadboard and placing it in its final position; **Step 2:** Installation of laser heatsink on the optical breadboard and fixing the laser system (VersaLase) on top of the heatsink; **Step 3:** Cutting of optical rails for corner mirrors and two reflecting mirrors and installation of the rail system onto the optical breadboard; **Step 4:** Installation of the pre-assembled acquisition chamber onto the corresponding rail; **Step 5:** Installation of the beam splitter (BS004, Thorlabs), which was placed into a cube adaptor (BS127CAM, Thorlabs), then fixed in place by a cage holder (CM1-4ER/M, Thorlabs) and finally mounted on a rail carrier; **Step 6:** Installation of all corner and laser reflecting mirrors; **Step 7:** Installation of the detection axis holder, infinity space tube, camera and its corresponding connection adapter units to the infinity space tube (U-CMAD3, U-TV1x-2 and U-TLU); **Step 8:** Installation of optical elements (beam expanders, telescope); **Step 9:** Installation of clean-up and emission filters; **Step 10:** Installation of Picard 4D stage in its correct position; **Step 11:** Connecting the controller boxes (Esio TTL controller box & VersaLase control box), VersaLase, Camera, USB 4D-stage and connecting them to the acquisition computer. The Versalase laser system was connected with the help of a controller box and a conventional RS-232 cable to the acquisition computer. ESio’s TTL controller box was connected directly to the VersaLase (not to the controller box) via individual SMB connector cables; **Step 12:** Setting up acquisition computer (installation of MicroManager and necessary plugins as well as drivers for VersaLase, Andor camera, Esio TTL controller box and pixel size calibration). The 5 second default security delay of the VersaLase laser system was disabled in the terminal of the Stradus VersaLase GUI software as described on the MicoManager website (https://micro-manager.org/wiki/Versalase); **Step 13:** Hardware configuration with Micromanager and testing for hardware recognition; **Step 14**: Final alignment of light-sheets by illuminating agarose containing fluorescent beads and/or stained specimens.

**OpenSPIM - alignment of the light-sheet for imaging**

The excitation light-sheets generated from each illumination path (left and right) were initially aligned using the 25 mm and 50 mm telescope lenses and the adjuster knobs of the two gimbal mounts of each corner mirror (Horizontal & Vertical). The light-sheets were visualized in a column of agarose within the water filled acquisition chamber and using the lowest laser power (1mW) (Supp. Figure 3, a and b). Additionally during the alignment emission filters and cylindrical lenses were removed. By adjusting the distance between the 25 and 50 mm telescope lenses and their distance to the illumination objective, the light-sheet first appears as an indistinct broad fuzzy beam crossing the field of view horizontally from left to right (Supp. Figure 3, A and C, orange arrows). The telescope lenses can then be further adjusted to increase the sharpness of the beam (step 1 in Supp. Figure 3, A) and to centre its focal point (step 2 in Supp. Figure 3, A). Next the horizontal gimbal mount adjuster knob is adjusted to bring the light-sheet in focus with the detection objective up to the point where it can be seen as a very thin stripe instead of a coarse beam. Finally the vertical gimbal mount adjuster knob is adjusted to center the light-sheet (step 3 in Supp. Figure 3, B). In this way both illumination paths are aligned and carefully centered until they overlap each other (Supp. Figure 3 D). By putting the cylindrical lenses and the emission filter back, the alignment of both excitation light-sheets can now be tested on fluorescent beads, which ideally homogenously cover the field of view and/or on a specimen embedded in agarose (Supp. Figure 3 E and F). Usually additional fine-tuning by adjusting the horizontal gimbal mount knob is necessary to achieve best imaging results.
